# Higher Cytokine and Opsonizing Antibody Production Induced by Bovine Serum Albumin (BSA)-Conjugated Tetrasaccharide Related to *Streptococcus pneumoniae* Type 3 Capsular Polysaccharide

**DOI:** 10.3389/fimmu.2020.578019

**Published:** 2020-12-04

**Authors:** Ekaterina A. Kurbatova, Nelli K. Akhmatova, Anton E. Zaytsev, Elina A. Akhmatova, Nadezhda B. Egorova, Natalya E. Yastrebova, Elena V. Sukhova, Dmitriy V. Yashunsky, Yury E. Tsvetkov, Nikolay E. Nifantiev

**Affiliations:** ^1^ Laboratory of Therapeutic Vaccines, Mechnikov Research Institute for Vaccines and Sera, Moscow, Russia; ^2^ Laboratory of Glycoconjugate Chemistry, N. D. Zelinsky Institute of Organic Chemistry, Russian Academy of Science, Moscow, Russia

**Keywords:** *Streptococcus pneumoniae* type 3, synthetic oligosaccharide, biotinylated oligosaccharide, conjugated oligosaccharide, antigen-binding capacity, vaccine, antibody, opsonophagocytosis

## Abstract

A number of studies have demonstrated the limited efficacy of *S. pneumoniae* type 3 capsular polysaccharide (CP) in the 13-valent pneumococcal conjugate vaccine against serotype 3 invasive pneumococcal diseases and carriage. Synthetic oligosaccharides (OSs) may provide an alternative to CPs for development of novel conjugated pneumococcal vaccines and diagnostic test systems. A comparative immunological study of di–, tri–, and tetra–bovine serum albumin (BSA) conjugates was performed. All oligosaccharides conjugated with biotin and immobilized on streptavidin-coated plates stimulated production of IL-1*α*, IL-2, IL-4, IL-5, IL-10, IFN*γ*, IL-17A, and TNFα, but not IL-6 and GM-CSF in monocultured mice splenocytes. The tetrasaccharide–biotin conjugate stimulated the highest levels of IL-4, IL-5, IL-10, and IFN*γ*, which regulate expression of specific immunoglobulin isotypes. The tetra–BSA conjugate adjuvanted with aluminum hydroxide elicited high levels of IgM, IgG1, IgG2a, and IgG2b antibodies (Abs). Anti-CP-induced Abs could only be measured using the biotinylated tetrasaccharide. The tetrasaccharide ligand possessed the highest binding capacity for anti-OS and antibacterial IgG Abs in immune sera. Sera to the tetra–BSA conjugate promoted greater phagocytosis of bacteria by neutrophils and monocytes than the CRM_197_-CP-antisera. Sera of mice immunized with the tetra–BSA conjugate exhibited the highest titer of anti-CP IgG1 Abs compared with sera of mice inoculated with the same doses of di– and tri–BSA conjugates. Upon intraperitoneal challenge with lethal doses of *S. pneumoniae* type 3, the tri– and tetra–BSA conjugates protected mice more significantly than the di–BSA conjugate. Therefore, it may be concluded that the tetrasaccharide ligand is an optimal candidate for development of a semi-synthetic vaccine against *S. pneumoniae* type 3 and diagnostic test systems.

## Introduction

Gram-positive, encapsulated, opportunistic bacteria *Streptococcus pneumoniae* are a major cause of upper and lower respiratory tract infection, meningitis, bacteremia, and acute otitis media. The chemical structure of pneumococcal capsular polysaccharide (CP) determines the serotype of *S. pneumoniae*. Among more than 90 serotypes of pneumococci, approximately 20 serotypes, including *S. pneumoniae* type 3, are clinically significant, and their CPs are used to prepare polysaccharide and conjugate pneumococcal vaccines.

*S. pneumoniae* serotype 3 isolates are predominant in the adult and elderly population (1–5), causing severe necrotic processes in the lungs (6, 7) and extrapulmonary localization (8–11). Diseases caused by *S. pneumoniae* serotype 3 are associated with high risk of mortality in children and adults (3, 12–19), thus causing global concern.

The capsule of *S. pneumoniae* serotype 3 has some unique characteristics (20). Greater capsule thickness and free CP (*i.e.*, not covalently linked to peptidoglycan) released during growth may explain the high virulence of *S. pneumoniae* serotype 3 and low efficacy of CP as a component of pneumococcal vaccines (21). A number of studies have demonstrated the limited efficacy of pneumococcal serotype 3 CP in the 13-valent pneumococcal conjugate vaccine against serotype 3 invasive pneumococcal diseases and carriage (22–25). Further improvement of the serotype 3-related component in the polyvalent pneumococcal vaccine is warranted. However, the immunologically insufficient bacterial CP obtained from culture fluid (26) cannot be easily substituted with a structurally more reliable, chemically synthesized product due to the difficult nature of polysaccharide synthesis (27).

Synthetic oligosaccharides (OSs) may provide an alternative to *S. pneumoniae* CPs for development of novel conjugate pneumococcal vaccines (28). Conjugates of synthetic di-, tri-, and tetrasaccharides related to CP of *S. pneumoniae* of serotype 3 equally protected mice from infection caused by this pneumococcal serotype (29). A hexasaccharide isolated from *S. pneumoniae* type 3 CP coupled to different proteins induced IgM and IgG antibodies (Abs) and provided protection against a mean lethal dose of *S. pneumoniae* type 3 in mice (30). In further studies, a synthetic tetrasaccharide hapten of the *S. pneumoniae* serotype 3 CP was chosen using glycan arrays. A tetrasaccharide–CRM_197_ conjugate reportedly induced anti-tetrasaccharide IgG Abs, promoted opsonophagocytosis, and protected against pneumonia caused by *S. pneumoniae* serotype 3 in mice (28, 31). Additionally, opsonizing Abs induced by glycoconjugates bound *S. pneumoniae* CPs and mediated host protection from pneumococcal infection (21, 32).

The recent study of tetanus toxoid (TT) conjugates of the synthetic penta-, hexa-, hepta-, and octasaccharide analogs of *S. pneumoniae* serotype 3 CP demonstrated the penta- and hexasaccharide conjugates to induce robust T cell-dependent IgG Ab responses in mice (33, 34). It was proved that immunization with the hexasaccharide-TT conjugate completely protected mice from *S. pneumoniae* serotype 3-caused infection and lung damage and significantly elongated mouse survival. Hexasaccharide-TT conjugate was identified as a particularly promising vaccine candidate against *S. pneumoniae* serotype 3 (34).

Here we present, for the first time, the results of an in-depth comparative study of the immunological properties of conjugated di-, tri-, and tetrasaccharides related to CP of *S. pneumoniae* serotype 3 aimed at selecting the most immunogenic synthetic oligosaccharide. We evaluated Th1/Th2/Th17 cytokine production by mononuclear cells of mice against biotinylated OSs immobilized on streptavidin-coated plates *in vitro*; OS conjugate-induced Abs isotypes; antigen-binding and opsonophagocytic capacity of glycoconjugate-induced Abs compared with Abs elicited by conjugated CP; and active protection of glycoconjugate-immunized mice upon challenge with *S. pneumoniae* serotype 3.

## Materials and Methods

### Synthetic Oligosaccharides and Their Conjugates

**Figure 1:**
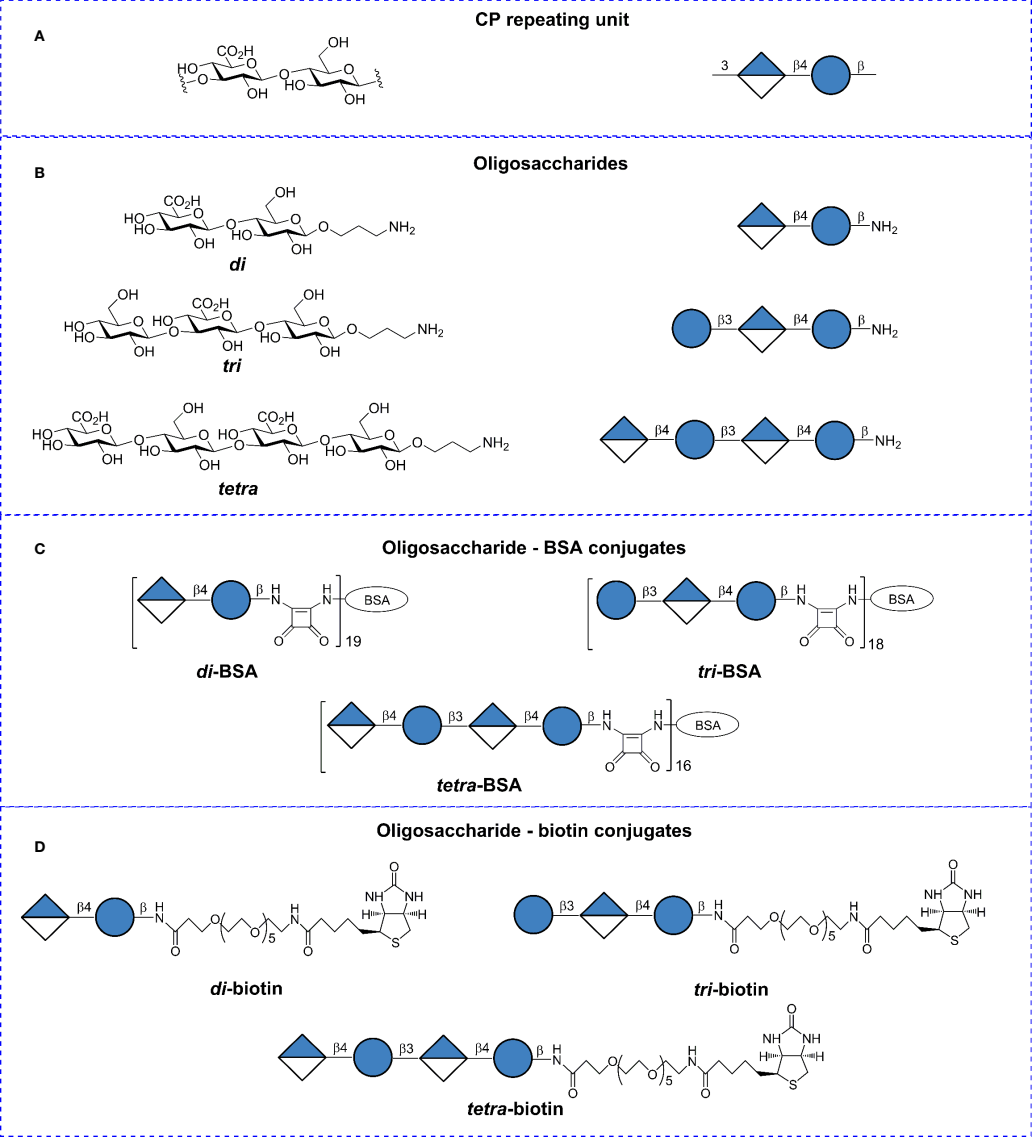
Structures of the *Streptococcus pneumoniae* type 3 capsular polysaccharide (CP), synthetic oligosaccharides (OSs), and glycoconjugates. **(A)** Repeating unit of the *S. pneumoniae* type 3 CP. **(B)** Synthetic spacer-armed OSs representing the CP-fragments. **(C)** BSA conjugates of synthetic OSs. **(D)** Biotin conjugates of synthetic OSs. Structural and symbolic representations are assigned according to symbol carbohydrate nomenclature for the carbohydrate sequences (38).

Synthetic OSs (35) were conjugated to bovine serum albumin (BSA) (Sigma-Aldrich, St. Louis, MO, USA) and biotin as previously described (36, 37). The structures and designations are illustrated in **Figure 1**. BSA has been frequently used as a protein carrier in engineered immunogenic glycoconjugates and other types of biomolecular systems (39). Matrix-assisted laser desorption ionization time-of-flight mass spectrometry data previously demonstrated that the di–, tri–, and tetra–BSA conjugates contained on average 19, 18, and 16 oligosaccharide ligands per protein molecule, corresponding with 9, 13, and 15% carbohydrate content by weight, respectively (35, 36). Lyophilized oligosaccharide–biotin and oligosaccharide–BSA conjugates are stable at +4°C without any decrease of activity within at least 3 years (observation period).

### Bacterial Capsular Polysaccharide

Bacterial CP was obtained from laboratory strain *S. pneumoniae* type 3 #10196 isolated on June 30, 2011 from blood of a child with bacteremia in the Microbiology Department of the “Scientific Center of Children’s Health”, Ministry of Health of the Russian Federation (Moscow, Russia). The strain was expanded in semisynthetic nutrient media. Isolation of CP was previously described (40). The presence of CP in the preparation was confirmed by NMR spectrometry.

### Animals

BALB/c male mice aged 6–8 weeks (n = 168) and two chinchilla rabbits weighing 2.5 kg were purchased from the Scientific and Production Centre for Biomedical Technologies (Moscow, Russia) and kept in the vivarium of the Mechnikov Research Institute for Vaccines and Sera. Housing, husbandry, blood sampling, and sacrificing conditions conformed to the European Union guidelines for the care and use of laboratory animals. Experimental designs were approved (Protocol # 2, February 12, 2019) by the Mechnikov Research Institute for Vaccines and Sera Ethics Committee.

### Biotinylated Oligosaccharide-Induced Cytokine Production

Quantitative determination of cytokines was performed as previously described (41–43). BALB/c male mice (n = 6) were sacrificed and spleens were aseptically removed and homogenized by passing through a sterile plastic strainer under aseptic conditions. After cells were centrifuged twice at 1,000 rpm for 5 min at 22°C, erythrocytes were lysed by a lysis buffer (0.15 M NH_4_Cl, 0.01 M NaHCO_3_, and 0.1 mM EDTA, pH 7.4) and then the cell pellets were washed twice with RPMI-1640 medium (Sigma-Aldrich). Further, cells were resuspended in complete RPMI-1640 medium (RPMI 1640 medium plus 10% fetal bovine serum, 2 mM glutamine, 100 IU/ml of penicillin and streptomycin, 15 mM HEPES, and 50 mM 2-mercaptoethanol) (Sigma-Aldrich). Cell counts were performed using a hemocytometer, and cell viability was determined using the trypan-blue (Sigma-Aldrich) dye exclusion technique with the results showing ≥95% viable cells. Cells were seeded into wells (2.5 × 10^5^ cells/well in complete RPMI-1640 medium) of streptavidin-coated plates (Thermo Fisher Scientific Inc., Waltham, MA, USA) pre-coated with biotinylated OSs at a binding capacity of 5 pmol biotin/well. OS–biotin conjugates were immobilized on the streptavidin-coated plates at 15 pmol/well. Results were compared with cells seeded into wells without pre-coating with biotinylated OS but stimulated by free OSs (10 μg/ml) or unstimulated (control). Purified CP of *S. pneumoniae* type 3 was not used as comparator because it may contain small amounts of bacterial impurities such as C-polysaccharide (teichoic acid) and other ligands to pathogen-associated pattern recognition receptors (PRRs). Cells were incubated for 24 h at 37°C in a humid chamber with a 5% CO_2_/95% air atmosphere. The supernatant was then collected and stored at −20°C until further quantification of cytokine levels using the FlowCytomix Mouse Thl/Th2 10-plex Kit test system containing beads coated with monoclonal antibodies to cytokines (IL-1*α*, IL-2, IL-4, IL-5, IL-6, IL-10, IL-17A, GM-CSF, IFN*γ*, and TNFα) following the manufacturer’s instructions (eBioscience, San Diego, CA, USA) using a FC-500 flow cytometer (Beckman Coulter, Brea, CA, USA).

### Immunization

A single dose of glycoconjugate ranged from 20 to 2.5 µg (carbohydrate content) in twofold dilutions with saline. Aluminum hydroxide [Sigma-Aldrich, 25 μl (250 μg) per immunizing dose] was added, and the mixtures were stored overnight at 4^°^C. Mice were immunized intraperitoneally with the di–, tri–, and tetra–BSA conjugates adjuvanted with aluminum hydroxide. Animals were dosed twice, on days 0 and 14 of the experiment. Similar immunization schedules were employed for doses of Prevnar 13 (Pfizer, New York, NY, USA) pneumococcal conjugate vaccine containing aluminum phosphate as an adjuvant, which comprised single doses of 1.1 µg per inoculation (equivalent to ½ the recommended dose for humans) and 2.2 µg per inoculation (equivalent the recommended dose for humans). The Prevnar 13 vaccine contains *S. pneumoniae* type 3 CP conjugated with inactive diphtheria toxin, CRM_197_, as the protein carrier. Mice injected with saline or aluminum hydroxide (250 µg) diluted in saline were used as controls. Antibacterial sera were recovered after repeated immunization of the rabbits with inactivated *S. pneumoniae* type 3 bacteria.

### Measurement of Antibody Response to Oligosaccharides and Capsular Polysaccharide

**Figure 2:**
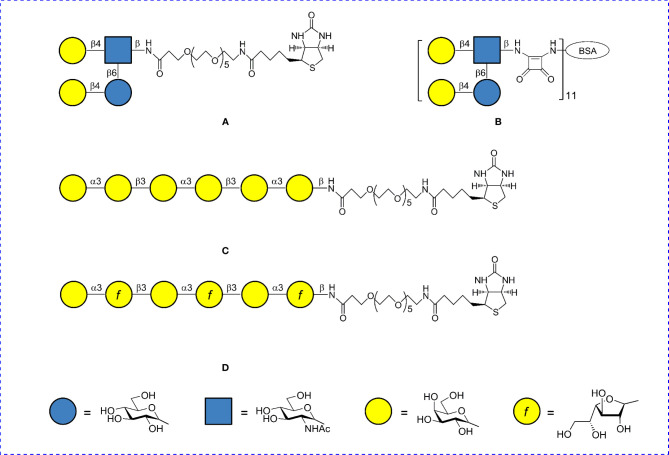
Structures of oligosaccharide conjugates used as negative controls. **(A)** Biotin conjugate of the tetrasaccharide of *S. pneumoniae* type 14 CP. **(B)** BSA conjugate of the tetrasaccharide of *S. pneumoniae* type 14 CP. **(C)** Biotin conjugate of the hexasaccharide of *K. pneumoniae* galactan II. **(D)** Biotin conjugate of the hexasaccharide of *K. pneumoniae* galactan I.

Specificity of interactions of di–, tri– and tetra–biotin coating antigens with antibodies raised to di– tri– and tetra–BSA conjugates was evaluated in ELISA. Biotinylated di-, tri- and tetrasaccharide related to *S. pneumoniae* type 3 (35) served as a positive control. Structurally unrelated biotinylated tetrasaccharide of *S. pneumoniae* type 14 (36, 44) and hexasaccharide fragments of galactans I and II of the O-antigen of *Klebsiella pneumoniae* (**Figure 2**) (45–47) were used as a negative control. The biotinylated oligosaccharides were immobilized on the surface of streptavidin pre-coated plates and sera to di–, tri–, and tetra–BSA conjugates were added.

Additionally, specificity of biotinylated di-, tri- and tetrasaccharide related to *S. pneumoniae* type 3 was estimated by comparison of their interaction with the rabbit hyperimmune serum to *S. pneumoniae* type 3 and sera to unrelated *S. pneumoniae* serotypes 1, 2, 4, 6A, 6B, 14, 19F as well as to *K. pneumoniae* O1K16, *Haemophilus influenzae* type b, *Escherichia coli* K-87, and *Staphylococcus aureus*. The sera were obtained by multiple immunizations of rabbits with inactivated bacterial cells.

Ab titers were measured by enzyme-linked immunosorbent assay (ELISA) from pooled sera of six mice/conjugate, or from individual blood, samples of six mice/dose as previously described (44, 46–48). Antibody titers to OSs of glycoconjugate-immunized mice were detected on the streptavidin-coated 96-well plates. ELISA assays were performed according to the manufacturer’s instructions (Thermo Fisher Scientific Inc). Briefly, 150 nM solutions of each biotinylated OS diluted in PBS were transferred into the streptavidin-coated wells (100 μl/well). OS was incubated for 2 h with shaking (300 rpm) at 22°C. Each well was washed 3× with 200 μl of wash buffer (phosphate-buffered saline (PBS) (Sigma) supplemented with 0.05% Tween 20 (PanReac Applichem, Barcelona, Spain) and 0.1% BSA (Sigma)). Serial dilutions of antisera were prepared and added to each well. Plates were incubated for 30 min at 22°C. Each well was washed 3× with 200 μl of wash buffer. Secondary rabbit anti-mouse peroxidase-conjugated IgM (µ chain), IgG1 (gamma 1 chain), IgG2a (gamma 2a chain), IgG2b (gamma 2b chain), and IgG3 (gamma 3 chain) (Rockland Immunochemicals, Inc., Pottstown, PA, USA) Abs (100 μl) were added to each well. After 30 min of incubation with shaking (300 rpm) at 22°C, wells were washed 3× with 200 μl of wash buffer. Enzyme substrate aliquots (100 μl) were added, followed by incubation for 15 min at 22°C. Antibody titers to CP in post-immunization murine sera were measured using flat-bottom plates (Biochemical LTD, Moscow, Russia) coated with *S. pneumoniae* type 3 bacterial CP (0.5 μg/well). Optical densities (ODs) were determined using an iMark microplate absorbance reader (Bio-Rad, Osaka, Japan) at 450 nm. Antibody titers are expressed as dilution of serum or as log_10_ values.

### Antigen-Binding Capacity of Glycoconjugate-Induced Antibodies

To study the Ab-binding capacity in the sera of mice immunized with OS-conjugates, CP, or bacteria, biotinylated OSs were adsorbed on the streptavidin-coated 96-well plates. After adding immune antisera (90 µl/well), a concentration gradient of the OS ligands or bacterial CP in PBS (1–10 µg/well) was inoculated (10 µl/well) into the wells. Incubations with ligands and CP were carried out for 30 min at 20–22°C. Plates were washed 3× with 200 µl/well of PBS-Tween 20. Next, working dilutions of peroxidase-conjugated rabbit anti-mouse IgG1 Abs (Rockland Immunochemicals) or goat anti-rabbit peroxidase-conjugated IgG Abs (Thermo Fisher Scientific) were added, as appropriate. Plates were incubated for 30 min at 22°C and then washed 3× with 200 µl/well of PBS-Tween 20. Next, 100 µl/well of TMB was added to stain the bound reaction products. After 15 min, the reactions were quenched with 1 M H_2_SO_4_. When CP was used as the wells’ coating antigen of flat-bottom plates (Biochemical LTD, Moscow, Russia), the reaction was carried out according to the same scheme, but incubation with ligands was at 37°C for 1 h and with secondary antibodies for 45 min at 37°C. The following procedures were as described above. ODs were determined at 450 nm with the iMark microplate absorbance reader. The results were presented as 50% inhibitory concentration (IC_50_) values, *i.e.*, the inhibitor concentration that led to a twofold OD decrease, and were calculated using calibration curves.

### Opsonophagocytosis Assay

Opsonophagocytosis was performed using flow-cytometry assay (49). The opsonizing activities of Abs elicited against the glycoconjugates were quantified by measuring the uptake of acetone-inactivated *S. pneumoniae* type 3 bacterial cells by neutrophils and monocytes in the peripheral blood of untreated mice. Inactivated bacteria were labeled by fluorescein isothiocyanate (FITC). FITC-labeled bacteria (10^9^ cells/ml) were treated with either sera (10 μl) from non-immunized mice, antisera (10 μl) from mice (n = 6 for each glycoconjugate) vaccinated twice with each of the synthetic conjugates (20 μg of carbohydrate), or CRM_197_–CP conjugate (1.1 μg of carbohydrate). Either (1) inactivated FITC-labeled bacteria, (2) inactivated FITC-labeled bacteria treated with non-immune sera, or (3) inactivated FITC-labeled bacteria treated with antisera to each BSA conjugate were added to peripheral blood from non-immunized mice (n = 10). Sera and antisera were added to the bacteria at a 1:1 ratio and then incubated for 20 min at 37°C, followed by washing by centrifugation (2,500 rpm, 10 min, 22°C) in RPMI-1640 medium. The number of neutrophils and monocytes that internalized FITC-labeled bacteria was determined using the Cytomics FC-500 flow cytometer with CXP software (Beckman Coulter). Cell population gates were determined by front and side light scattering and cell size; there were 10,000 cells per gate. The results were presented as the percentage of neutrophils or monocytes that phagocytized inactivated FITC-labeled *S. pneumoniae* type 3 cells.

### Glycoconjugate-Induced Active Protection in Immunized Mice

BALB/c mice were immunized intraperitoneally with each glycoconjugate on days 0 and 14 (n = 8 mice/dose). The same animals were challenged intraperitoneally after 2 weeks with 10^5^ colony forming units of *S. pneumoniae* type 3/0.5 ml. Non-immunized control mice (eight animals per group) were also challenged with the bacteria. Reference agent Prevnar 13 was given to mice (n = 6) at a single dose of 1.1 µg of CP *S. pneumoniae* type 3, respectively, and injected according to the same schedule as described above. The BSA conjugate of the tetrasaccharide related to CP of S. *pneumoniae* type 14 (**Figure 2B**) injected at a dose of 10 µg/mouse and adjuvanted with aluminum hydroxide was taken as a negative control. Mortality rates were calculated at 16 day post-infection.

### Statistical Analysis

Groups were compared using Mann–Whitney rank sum tests for independent samples. Fisher exact tests were used to evaluate mice survival after pneumococcal challenge. *P* values ≤0.05 were considered statistically significant using Statistica data analysis software system version 10 (StatSoft Inc., Tulsa, OK, USA).

## Results

### Cytokine Production Induced by Biotinylated Oligosaccharides

**Figure 3:**
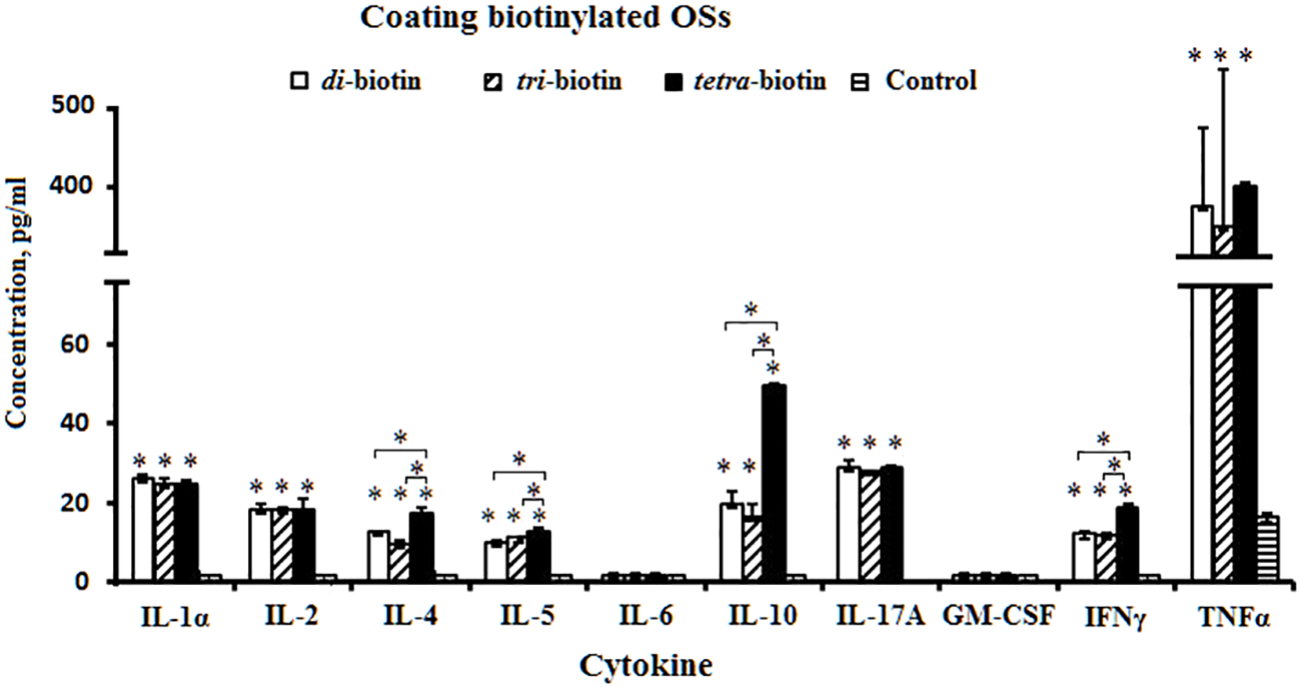
Levels of cytokine formation induced by biotinylated di-, tri-, and tetrasaccharides related to CP of *S. pneumoniae* type 3. The bars represent cytokine concentrations of triplicate evaluations of samples from mice (n = 6) in each group. Data are presented as a mean value ± SD. Mann–Whitney Rank Sum tests were used to determine significance between control and designated groups. *P < 0.05.

Mononuclear cells isolated from mice spleen were stimulated by biotin conjugates of the di-, tri-, and tetrasaccharides preimmobilized on streptavidin plates. Production of Th1/Th2/Th17 cytokines by the mononuclear cells was compared with the controls (free OSs stimulated or unstimulated cells). All biotinylated OSs stimulated production of IL-1*α*, IL-2, IL-4, IL-5, IL-10, IFN*γ*, IL-17A, and TNFα compared to the control (*P* < 0.05), but failed to induce production of IL-6 and GM-CSF (**Figure 3**). Free OSs (data not shown) and unstimulated cells failed to induce cytokine production.

Levels of IL-1*α*, IL-2, IL-17A, and TNFα did not differ upon action by the biotinylated di-, tri-, and tetrasaccharides. The tetrasaccharide–biotin conjugate stimulated the highest levels of IL-4, IL-5, IL-10, and IFN*γ* compared with biotinylated di- and trisaccharides with shorter chain lengths (*P* < 0.05). IL-4, IL-5, IL-10, and IFN*γ* have been shown to regulate the expression of specific immunoglobulin isotypes (50).

### Antibodies Isotypes Induced by Glycoconjugates

**Figure 4:**
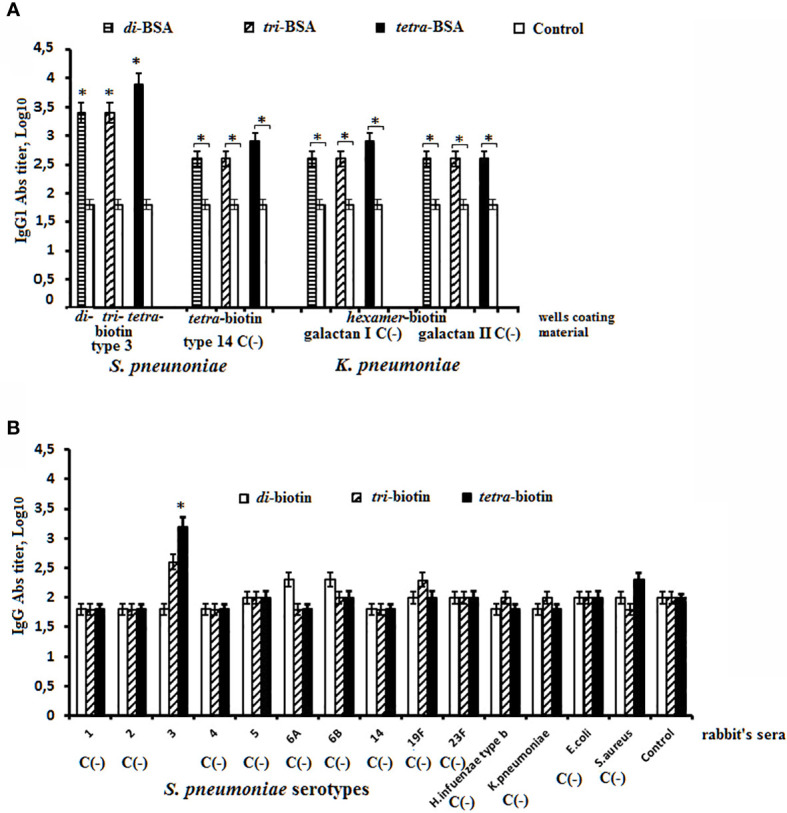
Specificity of biotinylated OSs related to *S. pneumoniae* type 3 in ELISA. **(A)** Sera to *S. pneumoniae* type 3 di–, tri–, and tetra–BSA conjugates were obtained after prime-boost immunization of mice with glycoconjugates adjuvanted with aluminum hydroxide. Abs titers were determined against *S. pneumoniae* type 3 biotinylated di-, tri-, and tetrasaccharide. Biotinylated oligosaccharides related to CP of *S.pneumoniae* type 14 and *K. pneumoniae* immobilized on the surface of streptavidin pre-coated plates were used as the negative control C(−). **(B)** The antibody titers against *S. pneumoniae* type 3 biotinylated di-, tri-, and tetrasaccharide were determined in sera to the unrelated serotypes of *S. pneumoniae* and some gram-negative and gram-positive bacteria. Antibody titers were transformed to log_10_ (3 assays per antiserum). The sera obtained from non-immunized mice were used as the control. Data are displayed as a mean value ± SD. Mann–Whitney Rank Sum tests were used to determine significance, *P < 0.05.

Two approaches were used to assess the specificity of interaction of biotinylated oligosaccharides related to CP of *S. pneumoniae* type 3 with immune sera in ELISA (**Figure 4**). In the first one, biotinylated oligosaccharides related to the heterologous *S. pneumoniae* type 14 and *K. pneumoniae* were used as negative controls (**Figure 4A**). Sera of non-immunized mice served as a control. To the biotinylated oligosaccharides immobilized on the streptavidin pre-coated plate, sera of mice immunized with the di–, tri–, and tetra–BSA conjugates related to *S. pneumoniae* type 3 adjuvanted with aluminum hydroxide were added. The antibody titer in the sera of mice immunized with *S. pneumoniae* type 3 BSA conjugates against homologous biotinylated oligosaccharides was higher than that against unrelated to *S. pneumoniae* type 3 biotinylated oligosaccharides in the negative control. In the control sera of non-immunized mice, the antibodies titer was the lowest (log_10_ < 2.0) against all biotinylated oligosaccharides. However, the antibodies’ titer against biotinylated oligosaccharides unrelated to *S. pneumoniae* type 3 in all cases was higher than in the sera of non-immunized mice. In the second approach, hyperimmune rabbit sera to *S. pneumoniae* types 1, 2, 3, 4, 5, 6A, 6B, 14, 19F, 23F, *K. pneumoniae*, *H. influenzae*, *E. coli*, and *S. aureus* were added to biotinylated oligosaccharides related to CP of *S. pneumoniae* type 3 (**Figure 4B**). We revealed a high titer of IgG antibodies in the sera to *S. pneumoniae* type 3 against biotinylated tetrasaccharide with no cross-reactions in the sera to the heterologous bacteria. The biotinylated di- and trisaccharide showed no diagnostic significance. The obtained results confirmed specificity of the biotinylated tetrasaccharide related to CP of *S. pneumoniae* type 3 in reactions with homologous antisera.

**Figure 5:**
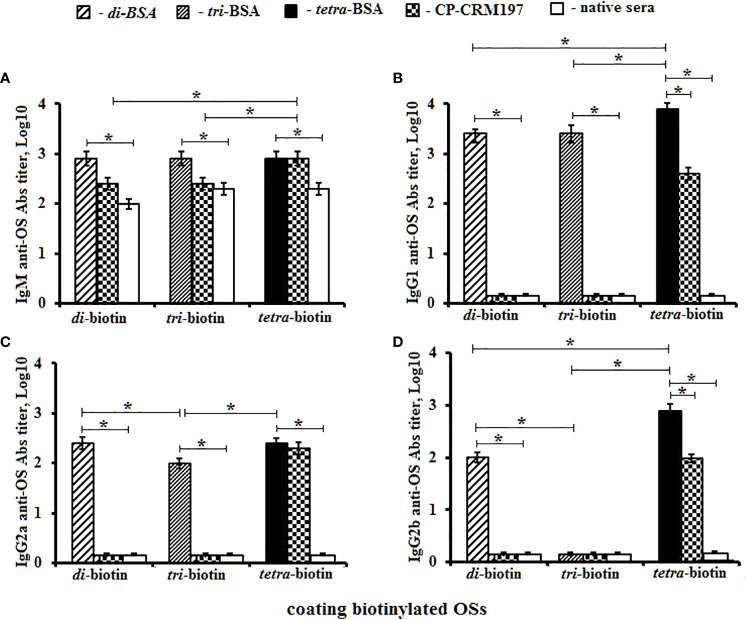
Anti-OS antibody (Ab) classes in pooled sera of BALB/C mice (n = 6) immunized with OS–BSA and CP–CRM_197_ conjugates. The biotinylated OSs (di–, tri–, and tetra–biotin) were applied as coating antigens. **(A)** IgM anti-OS Abs. **(B)** IgG1 anti-OS Abs. **(C)** IgG2a anti-OS Abs. **(D)** IgG2b anti-OS Abs. IgG3 level was low (<1:100) and not presented (three assays per antiserum). Ab titers were transformed to log_10_. Data are displayed as a mean value ± SD. Mann–Whitney Rank Sum tests were used to determine significance. *P < 0.05.

Titers of IgM, IgG1, IgG2a, IgG2b and IgG3 Abs were measured against biotinylated OSs in the pooled sera of glycoconjugate-immunized mice (**Figure 5**). Biotinylated OSs (di–, tri–, and tetra–biotin) were immobilized on streptavidin-coated plates to avoid interactions with antibodies specific to the protein carrier in the BSA conjugates.

Titers of IgM anti-OS Abs in sera of mice immunized with di–, tri–, and tetra–BSA conjugates did not differ from their corresponding biotinylated OSs and were higher than in native mice sera (*P* < 0.05) **(** **Figure 5А**). The highest titer of CP–CRM_197_-induced IgM Abs was determined against the biotinylated tetrasaccharide (*P* < 0.05).

The highest level of IgG1 anti-OS Abs was observed against the biotinylated tetrasaccharide in the sera of mice immunized with the tetra–BSA conjugate (log_10_ 3.9), compared to native serum and sera of mice inoculated with di–BSA (log_10_ 3.4) and tri–BSA (log_10_ 3.4) tested against biotinylated di- and trisaccharides, respectively (*P* < 0.05) **(** **Figure 5B**). Abs induced by the CP–CRM_197_ conjugate was not detectable using biotinylated di- and trisaccharides. Only the biotinylated tetrasaccharide was suitable for detection of anti-CP–CRM_197_-induced IgG1 Abs; however, its level was lower than that observed for tetra–BSA-induced Abs (*P* < 0.05).

Higher titers of IgG2a anti-OS Abs in the sera of mice inoculated with di–, tri–, and tetra–BSA conjugates were measured against corresponding biotinylated OSs in native mice sera (*P* < 0.05). The same levels of IgG2a anti-OS Abs were observed in sera of mice immunized with di- and tetra–BSA conjugates against biotinylated di- and tetrasaccharides, respectively (both log_10_ 2.4), which exceeded the titer (log_10_ 2.0) in sera of mice immunized with the tri–BSA conjugate (*P* < 0.05) **(** **Figure 5C**). Titer of Abs generated against the CP-CRM_197_ conjugate was not observed using biotinylated di- and trisaccharides (*P* < 0.05). Only the biotinylated tetrasaccharide was able to detect anti-CP-CRM_197_-induced IgG2a Abs.

Higher titers of IgG2b anti-OS Abs in the sera of mice immunized with di– and tetra–BSA conjugates were measured against corresponding biotinylated OSs in native mice sera (*P* < 0.05). Levels of IgG2b anti-OS Abs were higher in mice immunized with tetra–BSA and measured against biotinylated tetrasaccharide (log_10_ 2.9) compared with the sera of mice inoculated with di–BSA and measured against biotinylated disaccharide (log_10_ 2.0) (*P* < 0.05) (**Figure 5D**). Titer anti-OS Abs in the sera of mice immunized with tri–BSA conjugate was not detectable against di– and tri–biotin conjugates. Abs generated against the CP–CRM_197_ conjugate were only observed using the biotinylated tetrasaccharide; however, the level was lower than that of tetra–BSA-induced IgG2b Abs (*P* < 0.05). IgG3 Abs were undetectable.

**Figure 6:**
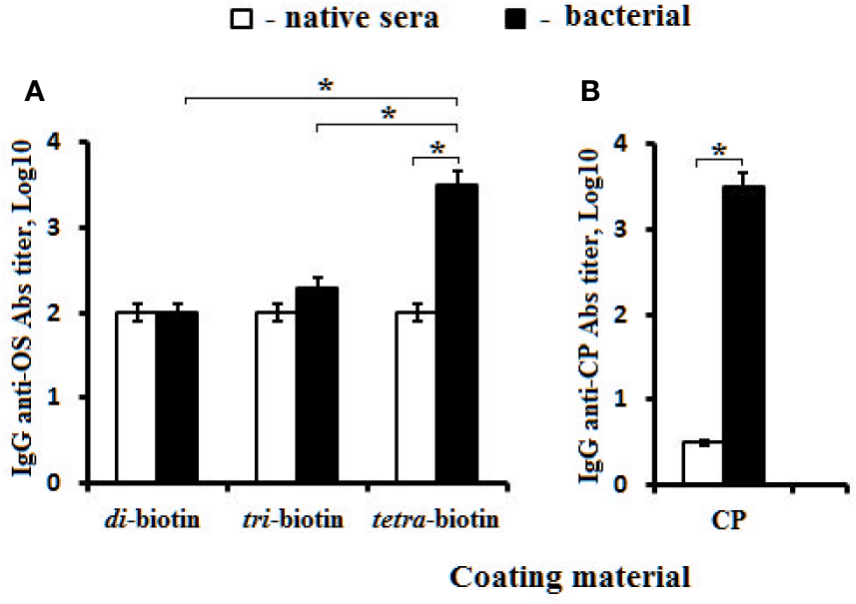
Antibodies (Abs) in pooled rabbit (n = 2) antibacterial sera. **(A)** Biotinylated OS di–, tri–, and tetra–biotin applied as the coating antigens. **(B)** CP used as the coating material. Antibody titers were transformed to log_10_ (three assays per antiserum). Data are displayed as a mean value ± SD. Mann–Whitney Rank Sum tests were used to determine significance, *P < 0.05.

The obtained data were confirmed using biotinylated OSs to reveal IgG Abs in the rabbit antibacterial sera (**Figure 6**). Biotinylated di- and trisaccharides were not able to bind anti-CP IgG Abs, and exhibited no difference from levels of IgG Abs in native rabbit sera (**Figure 6A**). The tetrasaccharide–biotin conjugate detected the highest titer of Abs (log_10_ 3.5) compared with the di– and tri–biotin conjugates; the level of IgG Abs detected in the antibacterial sera with tetra–biotin was significantly higher than that in native serum (*P* < 0.05). In the same rabbit sera, CP used as the well coating material possessed equal capacity to reveal anti-CP IgG Abs (Log_10_ 3.5) (**Figure 6B**) as the biotinylated tetrasaccharide.

Thus, the highest levels of IgG1, IgG2a, and IgG2b isotype Abs presented in the sera of mice immunized with the tetra–BSA conjugate. Abs generated against the CP–CRM_197_ conjugate and bacteria were only observed using the biotinylated tetrasaccharide.

### Anti-Capsular Polysaccharide Antibody Response Induced by Glycoconjugates

**Figure 7:**
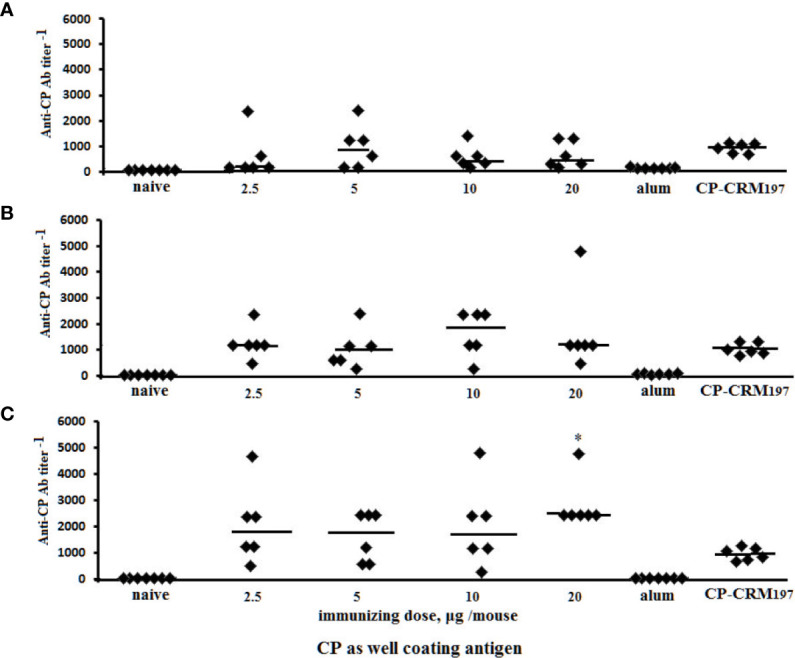
Anti-CP IgG1 antibody (Ab) titers in BALB/c mice (n = 6) immunized intraperitoneally with di–BSA **(A)**, tri–BSA **(B)**, and tetra–BSA **(C)** conjugates adjuvanted with aluminum hydroxide. Controls: naïve mice (n = 18); mice (n = 6) injected with aluminum hydroxide (250 µg) diluted in saline (three assays per each sera); mice (n = 6) received CP–CRM_197_ conjugate, 1.1 µg (three assays per each sera). Data represent individual anti-CP IgG1 Ab titers induced by glycoconjugates, bars indicate median ± SD. Mann–Whitney Rank Sum tests used to evaluate significance between the titer of Ab in anti-tetra–BSA compared with di– and tri–BSA antisera obtained from the mice immunized with 20 µg of the neoglycoconjugates, *P < 0.05.

Post-immunization anti-CP IgG1 Ab titers were determined in individual murine blood sera by ELISA using *S. pneumoniae* type 3 CP as the coating antigen (**Figure 7**). Ab level was not dependent on the immunizing dose of the glycoconjugates; however, titers of Abs to the tetrasaccharide–BSA conjugate in five of six mice were at the same elevated level. In the sera of mice immunized with CP–CRM_197_, the anti-CP IgG1 Ab titers increased but to a lesser extent than after the use of tetrasaccharide–BSA conjugate.

### Antigen-Binding Capacity of Glycoconjugate-Induced Antibodies

**Figure 8:**
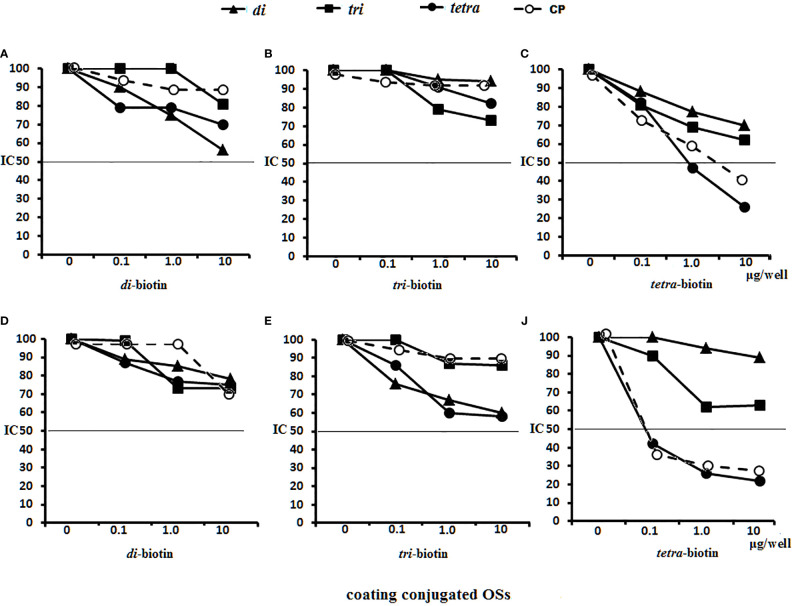
Inhibition of anti-OSs IgG1 antibodies (Abs) in mice and rabbit immune sera with OS ligands and CP. **(A**–**C)** Inhibition of IgG1 Abs in the pooled sera of BALB/c mice (n = 6 for each glycoconjugate) **(A)** Di–BSA conjugate antiserum in 1:500 dilution was tested against di–biotin capture material. **(B)** Tri–BSA conjugate antiserum in 1:500 dilution was tested against tri–biotin capture material. **(C)** Tetra–BSA conjugate antiserum in 1:1,000 dilution was tested against tetra–biotin capture material. **(D**–**F)** Inhibition of IgG Abs in the pooled sera of rabbits (n = 2) immunized with inactive *S. pneumoniae* type 3 cells. Dilution of sera tested against di–biotin **(D)** and tri–biotin coating material **(E)** was 1:500; tetra–biotin coating material **(F)** was 1:1,000, three assays per antiserum. Different antisera dilutions were used to reach approximately equal antibody level in tested sera samples. IC_50_—the half maximal inhibitory concentration.

Antigen-binding capacities of the Abs in the immune sera to OS-conjugates or bacteria were tested by ELISA using streptavidin plates coated with the biotinylated di-, tri-, and tetrasaccharides (**Figure 8**). Binding reactions were inhibited by adding the di-, tri-, and tetrasaccharide ligands and CP to the immune sera.

No inhibitory capacity was observed in the di–BSA antisera/di–biotin (**Figure 8A**) and tri–BSA antisera/tri–biotin (**Figure 8B**) systems, even to CP. In the tetra–BSA/tetra–biotin system, the tetrasaccharide ligand possessed the highest inhibitory capacity, including that of CP (**Figure 8C**). Thus, the tetrasaccharide ligand demonstrated maximal ability to inhibit binding between anti-OS IgG1 Abs and the immobilized biotinylated OS.

Neither the ligands nor CP inhibited interactions with di– and tri–biotin (**Figures 8D, E** **)** in the rabbit antibacterial sera. In the rabbit antisera/tetra–biotin system, the tetrasaccharide ligand and CP possessed the highest inhibitory activities (**Figure 8F**). Thus, the tetrasaccharide ligand and CP demonstrated comparable abilities to inhibit binding between antibacterial sera-IgG Abs and bound biotinylated tetrasaccharide.

**Figure 9:**
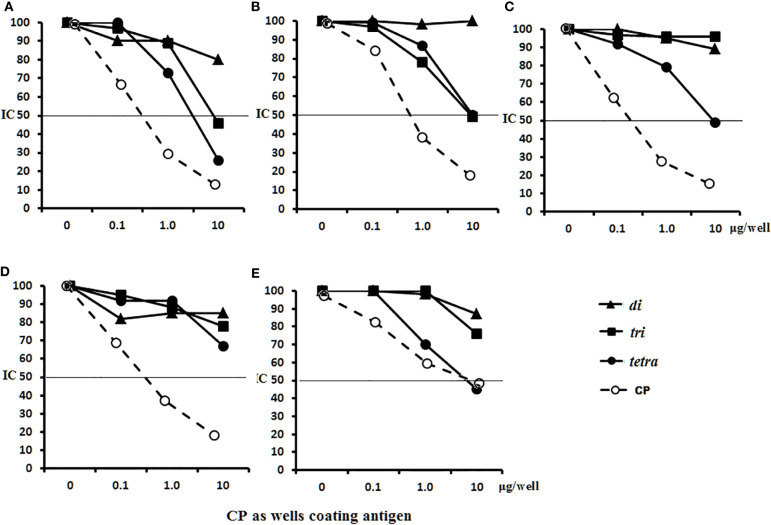
Inhibition of IgG1 antibodies (Abs) recognizing CP of *S. pneumoniae* type 3 as a coating antigen with OS ligands and CP in immune sera. The horizontal line indicates IC_50_ at the point of intersection of the inhibition curves. **(A)** Inhibition of IgG1 Abs in di–BSA conjugate sera in 1:400 dilution. **(B)** Inhibition of IgG1 Abs in tri–BSA conjugate sera in 1:800 dilution. **(C)** Inhibition of IgG1 Abs in tetra–BSA conjugate sera in 1:3,000 dilution. **(D)** Inhibition of IgG1 Abs in the pooled sera of BALB/c mice (n = 6) immunized with Prevnar 13 and *Streptococcus pneumoniae* type 3 CP at 1:400 dilution. **(E)** Inhibition of IgG Abs in the pooled sera of rabbits (n = 2) immunized with inactive *S. pneumoniae* type 3 cells at 1:2,500 dilution, three assays per antiserum. Different antisera dilutions were used to reach approximately equal antibody level in tested sera samples. IC_50_ — the half maximal inhibitory concentration.

The abilities of the OS ligands and CP to inhibit binding of IgG1 Abs to CP coating antigens either in glycoconjugate-induced sera or in antibacterial sera was studied (**Figure 9**). For sera of mice immunized with di–BSA (**Figure 9A**), tri–BSA (**Figure 9B**) and tetra–BSA conjugates (**Figure 9C**), the tetrasacchride ligand and CP possessed the highest inhibitory activities. The trisaccharide ligand exhibited inhibitory activity in the sera to di–BSA (**Figure 9A**) and tri–BSA conjugates (**Figure 9B**). The disaccharide ligand did not demonstrate any inhibitory activity. None of the OSs possessed inhibitory capacity in the sera of mice immunized with *S. pneumoniae* type 3 CRM_197_–CP (Prevnar 13) (**Figure 9D**). Only the tetrasaccharide ligand and CP reached IC_50_ for inhibiting interactions in antibacterial sera (**Figure 9E**).

In general, these data provide clear evidence that the tetrasaccharide ligand possessed maximal capacity to bind anti-OS and antibacterial Abs, without binding to CRM_197_–CP Abs in the sera.

### Opsonophagocytic Capacity of Glycoconjugate-Induced Sera

**Figure 10:**
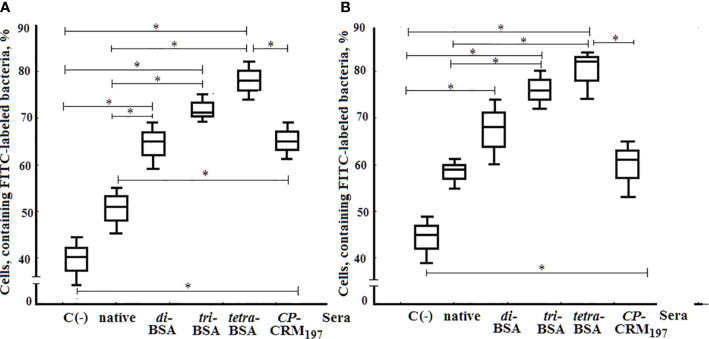
Opsonophagocytosis observed in pooled peripheral blood samples from non-immunized BALB/c mice (n = 10) after addition of inactivated FITC-labeled *S. pneumoniae* type 3 (C−), negative control; FITC-labeled bacteria with native serum; FITC-labeled bacteria treated with antisera obtained by immunization of mice with di–, tri–, and tetra–BSA conjugates adjuvanted with aluminum hydroxide and CP of *S. pneumoniae* type 3 CRM_197_ conjugate adjuvanted with aluminum phosphate. 3 assays per sample. **(A)** Neutrophils measured by flow cytometry. **(B;)** Monocytes measured by flow cytometry. Box and whisker plots represent the distribution of cells containing FITC-labeled *S. pneumoniae* type 3 bacteria. The box represents 25–75% distribution; the enclosed line is the median, the whiskers indicate the range from 2.5 to 97.5%. Mann–Whitney Rank Sum tests were used to calculate significance, *P < 0.05.

Opsonophagocytosis rates of *S. pneumoniaе* type 3 bacteria killed by neutrophils and monocytes collected from murine peripheral blood samples were examined by flow cytometry (**Figure 10**). The total number of active neutrophils that phagocytosed *S. pneumoniaе* type 3 bacteria was significantly increased in samples exposed to OS-conjugates and CRM_197_–CP antisera compared with control samples without sera (С−), or samples supplemented with native sera (*P* < 0.05). The opsonizing rates of the antisera to the glycoconjugates did not differ. Similarly, greater phagocytosis of *S. pneumoniaе* type 3 bacterial cells by monocytes was observed in the presence of sera to the tri– and tetra–BSA conjugates compared with native serum or the control (С−; *P* < 0.05). The lowest phagocytic activities of monocytes were observed after incubating bacteria with di–BSA or CRM_197_–CP conjugate antisera; no significant difference was found between monocyte phagocytosis rates of bacteria treated with di–BSA or CRM_197_–CP conjugates or in native serum. Only BSA–tetrasaccharide-induced antisera promoted greater opsonophagocytosis of bacteria cells by neutrophils (*P* < 0.05) and monocytes (*P* < 0.05) compared with antisera to CRM_197_–CP.

### Active Protection Upon Challenge of Glycoconjugate-Immunized Mice

The protective activities of glycoconjugates in mice challenged with *S. pneumoniae* type 3 are shown in **Table 1**. Highest protection levels from infection compared with the control group were induced by all doses tested of the tri- and tetra–BSA conjugates adjuvanted with aluminum hydroxide (*Р* < 0.01), whereas the di–BSA conjugate applied at 2.5, 5.0, and 20 µg doses was less effective (*Р* < 0.05). The *S. pneumoniae* type 3 CP–CRM_197_ conjugate adjuvanted with aluminum phosphate protected all mice from infection (*Р* < 0.01). The highest titer of IgG1 Abs to CP of *S. pneumoniae* type 3 was observed in the sera of mice immunized with 20 µg of tetra–BSA conjugate compared with mice inoculated with the same dose of di– and tri–BSA conjugates (*P < 0.05) or CP–CRM_197_ (*P < 0.05). The most immunologically active tetra–BSA conjugate at the dose of 2.5 µg/mouse induced approximately the same level of IgG1 antibodies (log_10_ 3.3) as CP–CRM_197_ *S. pneumoniae* type 3 in Prevnar 13 at the dose of 2.2 µg/mouse (log_10_ 2.9) measured in ELISA using CP *S.pneumoniae* type 3 as the well coating antigen. Injection of the unrelated *S. pneumoniae* type 14 tetra–BSA conjugate (**Figure 2B**) adjuvanted with aluminum hydroxide served as a negative control. Aluminum hydroxide alone did not protect the mice from infection.

**Table 1:** Protective activity of the glycoconjugates.

Immunogen	Single dose (carbohydrate content) per mouse, µg	IgG1 antibody titer, log_10_	Survivors	
			Day 0	Day 16
Di–bovine serum albumin (BSA)	2.5	2.5 ± 0.5	8	7*
	5.0	2.8 ± 0.5	8	7*
	10	2.7 ± 0.3	8	8**
	20	2.7 ± 0.3	8	7*
Tri–BSA	2.5	3.1 ± 0.2	8	8**
	5.0	3.0 ± 0.3	8	8**
	10	3.2 ± 0.4	8	8**
	20	3.2 ± 0.3	8	8**
Tetra–BSA	2.5	3.3 ± 0.3	8	8**
	5.0	3.2 ± 0.2	8	8**
	10	3.2 ± 0.4	8	8**
	20	3.5 ± 0,1*	8	8**
CRM_197_–CP S. *pneumoniae* type 3	1.1	2.9 ± 0.3	6	6**
	2.2	2.9 ± 0.2	ND	ND
BSA–tetrasaccha-ride S. *pneumoniae* type 14	10	<2.0	6	0
Aluminum hydroxide in saline	250	<2.0	8	2
Control	Saline	0 ± 0	8	1

CP of S. pneumoniae type 3 as the well-coating antigen in ELISA. ND, not determined. Difference between immunized mice and non-immunized mice (control). Fisher exact test, *P < 0.05, **P < 0.01.

Thus, all glycoconjugates adjuvanted with aluminum hydroxide possessed protective activity. The protective activities of the tri– and tetra–BSA conjugates were higher than that of the di–BSA conjugate.

## Discussion

*S. pneumoniae* type 3 CP comprises repeating units of *β*-(1, 3)-linked cellobiuronic acid (51) and is considered a T lymphocyte-independent type 2 (TI-2) antigen (52). Antibody response to TI-2 antigens is somehow influenced by T lymphocytes (53, 54). Human peripheral blood cells in response to TI-2 antigens reportedly upregulate IL-4 production *in vitro* (55). Another investigation demonstrated that TI-2 antigen stimulated IL-2, IL-4, and IFN*γ* production by T lymphocytes in murine spleen (56).

In our study under the action of biotinylated oligosaccharides related to CP of *S. pneumoniae* type 3 and immobilized on streptavidin-coated plates, splenocytes of non-immunized mice produced cytokines IL-1*α*, IL-2, IL-4, IL-5, IL-10, IFN*γ*, IL-17A, and TNFα in the culture medium. The biotinylated tetrasaccharide stimulated the highest production of IL-4, IL-5, IL-10, and IFN*γ* compared with the biotinylated di- and trisaccharides. It was surprising, since OSs are not Toll-like receptor (TLR) ligands and, being immobilized on the streptavidin-coated plates, could not be internalized by antigen-presenting cells (APC) and activate T cells followed by cytokine production. The absence of a ligand-receptor interaction between OS and PRRs was proved earlier using a synthetic hexasaccharide related to *S. pneumoniae* type 14 CP (40). The hexasaccharide ligand did not reveal the interaction with PRRs *in vitro* that was demonstrated using THP1-XBlue-CD14 cells expressing different TLRs and Nod-like receptors (NLRs). In contrast, stimulation of splenocytes with the di-, tri-, and tetrasaccharide ligands, which are available to the uptake by APC, failed to induce cytokine production in the supernatant (data not shown). The same result was obtained with short soluble synthetic glucans related to cell wall polysaccharides of the fungal pathogen *Aspergillus fumigatus*. Those glucans also failed to stimulate cytokine production by human polymorphonuclear cells *in vitro* despite their association with respective PRRs (41, 57–59).

We tried to explain a wide array of cytokines produced in the mice-donor splenocyte culture in response to the biotinylated OSs related to CP of *S. pneumoniae* type 3. It is known that the macrophage mannose receptor (MR) is able to bind purified CP from *S. pneumoniae via* carbohydrate recognition domains with following production of proinflammatory cytokines, such as IL-1, IL-6, TNFα and chemokines (60). C-type lectin SIGN-R1 expressed by macrophages, particularly in the marginal zone of the mouse spleen, binds streptococcal CPs from several different serotypes (60). The role of another macrophage receptor remains to be determined (61).

*γδ* T cells play specific roles in the immune response clearly separate from other classes of lymphocytes, having the ability to recognize unprocessed non-peptide antigens (62). Most of *γδ* T cells are found in the body’s barrier tissues and a little part in the blood and spleen of mice (40, 63). Certain subsets of *γδ* T cells express CD4 molecule. *γδ* T cells with a Th1 and Th2 phenotype produce IL-2, IL-4, IL-17A, IFN*γ*, and TNF. A great number of *γδ* T cell ligands are unknown yet. Probably, *γδ* T cells bind OS antigens without processing in APC in combination with the MHC-like CD1 molecule and activate the production of Th1/Th2 cytokines (62). The BSA conjugate with the *S. pneumoniae* type 3-related disaccharide adjuvanted with aluminum hydroxide augmented *in vivo* the level of *γδ* T cell surface molecule in spleen of mice from 0.6% before immunization to 19% after booster immunization (data not shown). Apparently, *γδ* T cells play a crucial role in the immune response to OS conjugates.

Summarizing our own results and data obtained by other authors, we suggested that soluble OS ligands can bind to a single carbohydrate recognition domain of the immunocytes without inducing cytokine production. Biotinylated OSs corresponding to the fragments of *S. pneumoniae* type 3 CP, being immobilized on the surface of the streptavidin-coated plates, likely acquired a spatial configuration and new properties that allow effective stimulation of splenocytes in culture medium with following production of different cytokines. Cytokine production level was directly proportional to OS length. This approach may be used for preliminary evaluation of the immune response to related CP.

Several T helper-derived cytokines, including IL-4, IL-5, IL-10, and IFN*γ*, have been shown to regulate expression of specific immunoglobulin isotypes. IL-4 and IL-5 switch IgM synthesis to IgG1, while IFN*γ* switches IgM synthesis to IgG2a (50). IL-10 can either enhance or suppress switching to particular murine immunoglobulin isotypes (64).

Nevertheless, the immunogenicity of TI-2 antigens is limited by their lack of direct recognition by T cells and their restricted ability to generate effective memory responses. Thus, CP or a synthetic OS-hapten related to CP is bound to a carrier protein to elicit a T-dependent IgG immune response (65–67).

The biotinylated oligosaccharides related to CP of *S. pneumoniae* type 3 displayed specificity in reactions with homologous antisera. The increase of the antibody titers in the sera to di–,tri–, and tetra–BSA conjugates against unrelated to *S. pneumoniae* type 3 biotinylated oligosaccharides may indicate the presence of common fragments in their structure that do not refer to protective epitopes. That fact was confirmed by the absence of protection of mice immunized with the *S. pneumoniae* type 14 tetra–BSA conjugate and challenged with *S. pneumoniae* type 3.

Di–, tri–, tetra–BSA conjugates and CP–CRM_197_ conjugate adjuvanted with aluminum salts induced IgM and IgG Abs isotypes. The tetra–BSA conjugate induced high levels of IgG1, IgG2a, and IgG2b Abs. The highest level of IgG1 Abs presented in the sera of mice immunized with the tetra–BSA conjugate adjuvanted with aluminum hydroxide, which is known to induce humoral (Th2) responses and Th1 cellular immunity (68). IgG1, IgG2a, and IgG2b classes of Abs generated against the CP–CRM_197_ conjugate were only revealed using the biotinylated tetrasaccharide. IgG3 Abs were not exhibited in the sera of mice immunized with OS- or CP-conjugates. IgG2a and IgG2b levels correlated best with opsonophagocytosis. The simultaneous presence of different IgG isotypes after immunization significantly increased the correlation between antibody levels and phagocytosis levels and thus protection against *S. pneumoniae* (69).

The tetrasaccharide ligand possessed maximal capacity to inhibit binding between anti-OS or antibacterial sera Abs and the bound biotinylated tetrasaccharide or CP. No differences in opsonophagocytic activity determined by flow-cytometry assay were observed between the glycoconjugates; only sera to the tetra–BSA conjugate caused greater phagocytosis of bacteria than Abs in sera to Prevenar-13, which includes CRM_197_-conjugated CP of *S. pneumoniae* type 3 as a component. The highest capacity of tetrasaccharide–BSA Ab to bind live bacteria of *S. pneumoniae* type 3 was observed in our previous study using the slide agglutination test (70).

Flow-cytometry opsonophagocytosis assay is rapid, reproducible, and specific and correlates well with opsonophagocytosis killing assay using HL-60 cell lines that is technically more difficult to use and require tissue culture facilities (71, 72). Thus, flow-cytometry opsonophagocytosis assay may be considered as a good alternative to the killing assay especially in research practice (71). According to our previous observations, flow-cytometry opsonophagocytosis assay produced reliable results and proved the highest efficacy of conjugated tetrasaccharide related to *S. pneumoniae* type 14 that coincided with other immunological tests (44). Flow-cytometric-based opsonophagocytosis is a perspective method and according to recent studies is used for rapid quantification of the opsonizing capacity of antigen specific antibodies elicited in response to immunization (73). Other modifications of opsonophagocytosis assays are used now (74–76).

The high virulence of S*. pneumoniae* serotype 3 strains allows their use in the study of experimental pneumococcal infection in animals (10, 77, 78). We revealed minimal differences in the protective activities of tri– and tetra–BSA conjugates, which both demonstrated highly protective properties. In recent studies, the tetrasaccharide–CRM_197_ conjugate elicited protection against pneumonia caused by *S. pneumoniae* 3 in mice in a lethal transnasal challenge model evidenced by opsonophagocytosis assays (31).

CP of *S. pneumoniae* type 3 possesses not only low immunogenicity in pneumococcal conjugate vaccines but also insufficient diagnostic capacity. Serotype 3 CP of the Luminex multiplex assay demonstrated inferior inter-laboratory reproducibility than other components with unreliable results (79). In the current study, only application of the biotinylated tetrasaccharide allowed determination of IgG Ab classes in the sera of mice immunized with Prevnar 13. This data indicates the reasonable use of the biotinylated tetrasaccharide for the development of novel diagnostic test systems for measuring Ab levels to CP of *S. pneumoniae* type 3.

## Conclusion

Conjugated tetrasaccharide, which is composed of two repeating units of CP of *S. pneumoniae* type 3, induced higher levels of pro-inflammatory and inflammatory cytokines compared with di- and trisaccharide conjugates, and elicited the expression of specific immunoglobulin isotypes. The tetrasaccharide ligand possessed maximal capacity to bind to anti-OS and antibacterial Abs. The conjugated tetrasaccharide protected mice upon lethal challenge with *S. pneumoniae* type 3 as evidenced by opsonophagocytosis assay. Only the biotinylated tetrasaccharide was able to detect CP–CRM_197_ conjugate-induced Abs. It may be concluded that the tetrasaccharide ligand is an optimal candidate for the development of a semi-synthetic vaccine against *S. pneumoniae* type 3 and can be applied for the improvement of existing and development of new diagnostic test systems. Commercial conjugate pneumococcal vaccines may be improved by adding the conjugated synthetic tetrasaccharide instead of low immunogenic CP of *S. pneumoniae* type 3.

## Data Availability Statement

The original contributions presented in the study are included in the article/supplementary material; further inquiries can be directed to the corresponding authors.

## Ethics Statement

The animal study was reviewed and approved by Ethics Committee of the Mechnikov Research Institute for Vaccines and Sera (Protocol # 2, February 12, 2019).

## Author Contributions

EK planned the study, summarized the results, and performed statistical analysis of the data. NA studied the cytokine production and opsonophagocytosis. AZ studied the isotypes and titer of antibodies in different test systems and the protective activity of glycoconjugates in mice. EA performed inhibition ELISA using different coating antigens and immune sera. NE compared the results with the data of contemporary literature. NY obtained the capsular polysaccharide and immune rabbit’s sera. ES performed the chemical synthesis of oligosaccharides. DY performed the OS conjugation with the protein carrier. YT performed the chemical analysis of synthetic OSs, neoglycoconjugates and bacterial capsular polysaccharide. NN planned the study, analyzed the results and compared them with the data of contemporary literature. All authors contributed to the article and approved the submitted version.

## Funding

This work was supported by the Russian Science Foundation (grant no. 19-73-30017).

## Conflict of Interest

The authors declare that the research was conducted in the absence of any commercial or financial relationships that could be construed as a potential conflict of interest.
